# Supplementary material for: Novel Models of Streptococcus canis Colonization and Disease Reveal Modest Contributions of M-Like (SCM) Protein
Source: Microorganisms. 2021 Jan 16;9(1):183. doi: 10.3390/microorganisms9010183 (PMC7829700; doi:10.3390/microorganisms9010183)
Supplement: Supplementary file 1 [file microorganisms-09-00183-s001.zip › Supp. Material.pdf]

Novel models of *Streptococcus canis* colonization and disease reveal modest contributions of M-like (SCM) protein

Ingrid Cornax, Jacob Zulk, Joshua Olson, Marcus Fulde, Victor Nizet, Kathryn A Patras

Supplementary Material

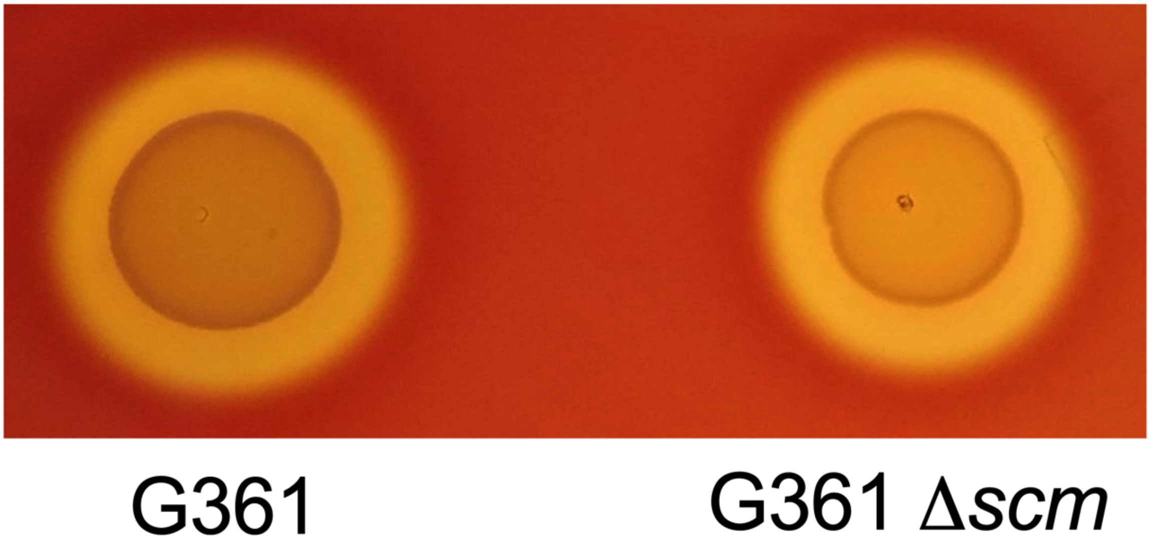

Supplemental Figure 1. SCM has no effect on hemolytic activity. Representative image of S. canis G361 or G361Δscm spotted on blood agar plates and grown for 24 h at 37°C.

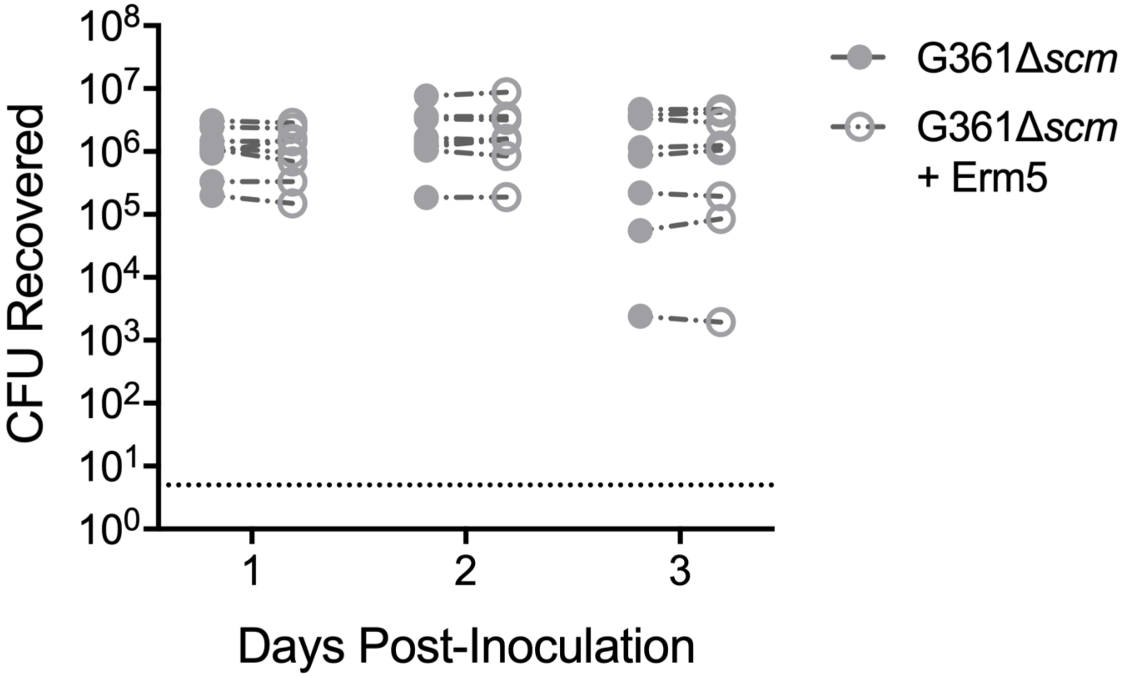

Supplemental Figure 2. SCM insertional mutagenesis is stable *in vivo*. CD1 female mice were vaginally administered 1×10^7 CFU of G361Δ*scm*. Mice were vaginally swabbed daily, and the levels of bacterial CFU recovered from swabs, plated on media with or without antibiotic selection are shown. Dotted line indicates limit of detection. Data were analyzed by Wilcoxon matched-pairs signed rank test and determined not significant.
